# The Co-occurrence of Specialty Vape Shops, Social Disadvantage, and Poor Air Quality in the United States: An Assessment of Cumulative Risks to Youth

**DOI:** 10.1089/heq.2021.0151

**Published:** 2022-02-25

**Authors:** P. Dilip Venugopal, Aura Lee Morse, Rudaina Alrefai-Kirkpatrick, Cindy Tworek, Hoshing W. Chang

**Affiliations:** Office of Science, Center for Tobacco Products, U.S. Food and Drug Administration, Beltsville, Maryland, USA.

**Keywords:** socioeconomic status, health equity, tobacco control, e-cigarettes, environmental justice, environmental health

## Abstract

**Introduction:** We conducted a cumulative environmental health risk assessment of whether specialty vape shops and poor air quality are more likely to co-occur in socially disadvantaged neighborhoods where racial/ethnic minority youth live.

**Methods:** We examined the population-adjusted incidence of specialty vape shops in relation to youth race/ethnicity, neighborhood socioeconomic status (SES), and air quality (nitrogen dioxide [NO_2_]) at the census tract level across the conterminous United States for 2018.

**Results:** We did not find disparity in vape shop incidence related to minority youth race/ethnicity. Vape shop incidence was significantly negatively associated with all the youth race/ethnicities examined. The two lowest SES quintiles had nearly double the rate of specialty vape shop incidence compared with the highest SES quintile. Specialty vape shop incidence increased with NO_2_ concentration, with more vape shops in poor air quality neighborhoods.

**Conclusions:** Specialty vape shops are disproportionately present in neighborhoods with poor air quality and where socially disadvantaged youth live. The increased incidence of vape shops in poor air quality neighborhoods, particularly in an urban context with increased traffic emissions, further points to potentially disproportionate impacts on disadvantaged populations due to cumulative social and environmental risks. This raises environmental justice and health equity concerns. Retailer-focused strategies aimed at limiting youth exposure to electronic cigarettes' labeling and advertising, preventing sales to minors, and limiting the number of retailers in low-SES neighborhoods may reduce initiation and help prevent tobacco-related health disparities among youth.

## Introduction

Socioeconomic status (SES) is an important risk factor for both tobacco use and tobacco-related disease, with a positive association between SES and health.^1–3^ Low-SES communities in the United States experience tobacco-related health disparities that worsen health status and shorten life expectancy.^1,3,4^ Inequities in exposure to tobacco marketing and advertising, exposure to environmental tobacco smoke, tobacco use, and access to cessation programs contribute to tobacco-related health disparities for low-SES communities.^1,3–8^

Of concern is that low-SES communities in the United States, particularly children and the elderly, are also disproportionately exposed to environmental hazards and their health impacts.^9–12^ Social stressors such as low-SES may interact with environmental exposure, worsening health outcomes.^11,13,14^ Communities of low SES or those with high proportions of racial/ethnic minorities tend to have increased social and economic vulnerabilities. Cumulative exposure to environmental hazards may cause greater harm to health than would likely occur in the absence of those vulnerabilities.^11,13,14^

Tobacco-related health disparities are a function of the interplay of individual, physical, and social factors. Therefore, cumulative risk assessments^13,15^ and other socioecological approaches^16^ may be particularly useful for understanding and addressing tobacco-related health disparities. Such approaches aid in identifying both the root causes of tobacco-related health disparities and the populations that are concurrently exposed to multiple social and environmental hazards.^3,13,14^

The role of the tobacco retail landscape in tobacco-related health disparities has received increased attention, including the opportunities it presents for tobacco control and eliminating disparities.^4,17^ For traditional tobacco products, studies suggest that retailers are more numerous and more dense in racial/ethnic minority and low-SES communities and are broadly associated with disparities in exposure to tobacco marketing, tobacco use, and tobacco-related diseases.^3,5,6,8,18^

Recent studies also suggest that the retail landscape for electronic nicotine delivery systems (henceforth, e-cigarettes), as with traditional tobacco retailers, poses environmental health risks to youth.^19–21^ Consistent with a trend over the past several years, data from the 2020 National Youth Tobacco Survey show that e-cigarette was the most commonly used tobacco product among youth attending school.^22^ Retailers specializing in tobacco products, such as vape shops, are among the most common sources of e-cigarettes for youth after social sources.^23,24^

E-cigarette retailer density and proximity are associated with higher exposure to e-cigarette advertisements and e-cigarette use among youth.^19,20^ Taken together, these studies suggest that vape shops are features of the built environment that can be a source of harm to health through youth exposure to tobacco product labeling and advertising. Therefore, vape shops are themselves associated with an environmental health risk to youth.

Available national assessments report disproportionately higher density of vape shops in socially disadvantaged^25^ and racial/ethnic minority neighborhoods.^21,25^ This is of concern as recent reports indicate that students from socially disadvantaged communities attending Title 1 schools (schools with high numbers or high percentages of students from low-income families) are susceptible to e-cigarette use.^26^

Hispanic high schoolers are more likely to be susceptible to e-cigarette use compared with their non-Hispanic counterparts.^27^ Among Hispanic youth, use prevalence was highest for e-cigarettes compared with other products. For non-Hispanic Black or African American youth, e-cigarette use prevalence (6.2%) is similar to use prevalence for cigars (6.5%), the tobacco product most commonly used by this population.^22^ These factors raise health concerns, given reports of respiratory symptoms in youth associated with e-cigarette use.^28–30^

These reports also raise environmental justice (EJ) concerns. Executive Order 12898 and the associated Council on Environmental Quality guidance direct federal agencies to identify and address EJ aspects of their activities. Following this, the Federal Interagency Working Group on EJ recommends that federal agencies consider the potential for environmental health hazards to disproportionately affect minority and low-income populations through the National Environmental Policy Act processes.^31^

Identifying and addressing disproportionately high and adverse human health and environmental effects on low-income populations and American Indian/Alaska Native communities are goals for the U.S. Department of Health and Human Services as part of strategic elements of EJ.^32^ Additionally, Executive Order 13045 encourages federal agencies to identify and assess environmental health and safety risks that may disproportionately affect children.

As nascent sources of exposure, vape shops are a feature of the built environment that can be a source of harm to health through exposure of youth to tobacco labeling and advertising, and e-cigarette retailers may contribute to the cumulative environmental health risks faced by low-SES and minority communities who already bear disproportionate impacts of tobacco-related health disparities.^1–3,13^

Little evidence exists regarding whether vape shops contribute to the cumulative social and environmental risks experienced by low-SES and minority youth. Such EJ assessments of the vape shop retail landscape, in relation to marginalized populations and other co-occurring environmental hazards, may aid efforts tackling health disparities from tobacco use among youth.^21^

In this study, we used a place-based approach to examine the co-occurrence of two environmental health risks—specialty vape shops and poor air quality—as well as a social stressor (SES). We further examined whether these factors co-occur in places where youth who belong to racial/ethnic minority communities live. Specifically, we examined the statistical relationships of the presence of specialty vape shops with youth race/ethnicity, a composite SES indicator (as a measure of social stress), and an ambient air quality indicator (as a measure of exposure to environmental hazards) at the census tract level across the conterminous United States.

## Methods

### Ethics approval and consent to participate

The study did not involve any human participants or personally identifiable information, and used secondary data collected from publicly available sources (e.g. Census bureau). Therefore, ethics approval and consent to participate were not applicable.

### Specialty vape shop data

A restricted definition of vape shops is appropriate for an accurate assessment of the socioenvironmental correlates of specialty vape shops. Tobacco retailers carrying a variety of product types outnumber specialty vape shops. In addition, the association of traditional tobacco retailers with minority and low-income communities is well established. Hence, a restrictive definition, excluding establishments that sell tobacco products other than e-cigarettes, helps reduce the potentially confounding influence of tobacco retailers carrying varied tobacco products.^21,33^

We utilized the national specialty vape shop data and methods that were employed for identifying and locating 7475 specialty vape shops throughout the conterminous United States for 2018 (Supplementary data file_specialtyvapeshops).^21^ We mapped the specialty vape shop location data from the study by Venugopal et al.^21^ to the 5-year estimates of the 2014–2018 American Community Survey geodatabase census tracts across the conterminous United States.

We derived the vape shop counts within 71,927 census tracts, excluding tracts with 100% water area or zero population and three vape shops, each located in zero-population tracts.

### Race/ethnicity data among youth

Racial/ethnic diversity is higher among youth, the population of interest examined in this study, compared with the overall U.S. population.^34^ Therefore, we used youth race/ethnicity data rather than data from the entire population. We obtained the census tract-level demographic data on age group/sex/race estimates from the Census Bureau's American Community Survey 5-year data profile tables (B01001 table series; 2014–2018).

We then compiled data on the number of persons below 18 years of age (henceforth, youth) by race/ethnicity of interest (White alone, not Hispanic or Latino; Black or African American alone; Hispanic or Latino; American Indian or Alaska Native alone; and Asian alone) and calculated the percentages in each census tract.

### SES as a social stressor

We used the composite SES index data available from the Surveillance, Epidemiology, and End Results census tract-level SES database.^35,36^ The SES index is derived from seven variables (percent working class population, percent adult unemployment, educational attainment, median household income, percent of population living below 150% of the national poverty line, median rent, and median home value).

The SES composite index is categorized in quintiles of equal population size, with the first quintile (Group 1) representing the lowest and the fifth quintile (Group 5) representing the highest SES.^36^ We obtained the census tract SES quintile data (2013–2017) and overlaid them with the vape shop counts and race/ethnicity data. Due to differences in SES composite index data availability at the census tract level, 102 of 7475 specialty vape shops (1.4%) in 2333 census tracts were excluded.

SES quintile data were integrated with vape shop counts and race/ethnicity data for 69,594 census tracts (Supplementary data file_Censustractsdata).

### Air quality indicator as an environmental hazard

We used nitrogen dioxide (NO_2_) as an environmental hazard indicator variable. NO_2_ is used as an overall indicator of air pollution and air quality, especially for traffic-related air pollutants.^37^ Long-term NO_2_ exposure is associated with increased incidence of pediatric asthma and respiratory infections.^37,38^

We utilized a global, high spatial resolution (0.01°×0.01°), annual average, NO_2_ surface concentration dataset^38,39^ for 2018. We used zonal statistics to aggregate and calculate median NO_2_ concentration (ppb) at each U.S. census tract.

### Statistical analyses

At the census tract level, we generated summary statistics of youth race/ethnicity and socioenvironmental attributes by the number of specialty vape shops. We generated summary statistics for both the broader dataset of vape shop counts by census tracts and the dataset used for final analyses. We first examined spatial autocorrelation in vape shop counts per census tract using Global Moran's *I* test statistic with a first-order queen contiguity spatial weight matrix, defining the neighborhood structure of each census tract.

We removed 17 census tracts without defined neighbors from the 69,594 tracts, one of which contained a single vape shop. We tested the statistical significance of Global Moran's *I* value using a permutation test (Monte Carlo simulation; 999 permutations) and generated a spatial lag variable.

We analyzed the statistical association of vape shop counts in the remaining 69,577 census tracts with youth race/ethnicity, SES, and median NO_2_ using a generalized linear mixed-effects model (GLMM). First, we ran multiple candidate models fitted using maximum likelihood and Laplace approximation. The candidate models contained vape shop count as a response variable; race/ethnicity, SES, and NO_2_ as predictor variables; natural log of census tract population as an offset variable; and spatial lag variable as a random effect to account for potential spatial autocorrelation.

Race/ethnicity data on White alone, Hispanic, or Latino youth were excluded from the analysis given the focus on examining census tract-level vape shop density associations with proportions of minority youth and to avoid multicollinearity. We ran Poisson, negative binomial, zero-inflated Poisson, zero-inflated negative binomial, and truncated Poisson hurdle or truncated negative binomial hurdle GLMMs, all with log link functions. We then selected the best-fit and parsimonious model based on the combination of Akaike information criteria and Bayesian information criteria, with lower values indicating better fit.

We used Wald *χ*^2^ tests to determine the statistical significance of the predictors in the selected model. We ran *post hoc* comparisons among SES quintiles using Dunnett's test with SES Group 5 (highest SES) as the control group and report the incidence rate ratios and their 95% confidence intervals.

We performed all spatial and statistical analyses using the *R* program.^40^ We used R packages, *arsenal*^41^ for summary statistics, *spdep*^42,43^ and *raster*^44^ for spatial analysis and overlays, *glmmTMB*^45^ for the GLMM, *multcomp*^46^ for the Dunnett test, and *performance*^47^ for checking multicollinearity. We extracted and plotted model estimates with the package *sjPlot.*^48^

## Results

### Summary statistics

We provide summary statistics of census tract-level youth race/ethnicity and socioenvironmental attributes by the number of specialty vape shops in Supplementary Table S1. Of the 69,577 census tracts used for the analysis, 91.71% had no specialty vape shops, 8.00% had one, 1.03% had two, 0.13% had three, and 0.03% had four or more specialty vape shops. Compared with census tracts with no vape shops, those with higher numbers of vape shops had a larger total population. Compared with census tracts with fewer vape shops, census tracts with more vape shops had higher numbers of youth who were Hispanic or Latino, Asian alone, or American Indian/Alaska Native alone, but lower numbers of youth who were Black or African American alone.

A higher proportion of SES Group 5 (highest SES) was found in census tracts with no vape shops than in census tracts with vape shops. Median NO_2_ concentrations were higher in census tracts with more vape shops, compared with census tracts without vape shops. In addition, the summary statistics of race/ethnicity and air quality data were similar between the final dataset used for statistical analysis and the larger vape shop counts at 71,927 census tracts (not shown).

### Specialty vape shops versus socioenvironmental factors

Global Moran's *I* test statistic and density plot of permutation outcomes (not shown) indicated significant global spatial autocorrelation in specialty vape shop counts in census tracts across the United States (Global Moran's *I*=0.044, *p*=0.001). As per model selection results, we analyzed the statistical association of vape shop counts at census tracts with race/ethnicity, SES, and median NO_2_ variables using a negative binomial GLMM (Supplementary Table S2). Model diagnostics did not raise multicollinearity concerns, as indicated by the variance inflation factors (range 1.02–1.33) and tolerance values (range 0.73–0.98) (Supplementary Table S3).

Among youth, specialty vape shop incidence was significantly negatively associated with all the race/ethnicity categories we examined, with overall weak strength of associations, as indicated by incidence rate ratios (Table 1). At the census tract level, the number of vape shops decreased in relation to the proportion of youth represented by each race/ethnicity category. We did not find disparity in vape shop incidence related to youth race/ethnicity.

**Table 1.** Association of Specialty Vape Shop Counts with Youth Race/Ethnicity and Socioenvironmental Attributes at the Census Tract Level Across the Conterminous United States, 2018

Predictors	Incidence rate ratios (95% CI)^a^	Wald χ^2^/***Z*** value	***p*** ^b^
Race/ethnicity of youth			
Black or African American alone	0.99 (0.99–0.99)	154.950	**<2.2e-16**
Asian alone	0.99 (0.98–0.99)	13.169	**0.0003**
American Indian/Alaska Native alone	1.00 (0.99–1.00)	1.855	0.1735
Hispanic or Latino	0.99 (0.99–0.99)	197.315	**<2.2e-16**
Socioeconomic status [control: Group 5 (highest socioeconomic status)]		280.2698	**<2.2e-16**
Socioeconomic status [Group 4]	1.60 (1.47–1.74)	11.11^c^	**<2.2e-16**
Socioeconomic status [Group 3]	1.78 (1.64–1.94)	13.44^c^	**<2.2e-16**
Socioeconomic status [Group 2]	1.98 (1.82–2.16)	15.66^c^	**<2.2e-16**
Socioeconomic status [Group 1 (lowest socioeconomic status)]	1.94 (1.77–2.13)	13.86^c^	**<2.2e-16**
Nitrogen dioxide concentration (median; ppm)	1.07 (1.06–1.07)	335.6670	**<2.2e-16**
Random effects			
σ^2^	2.10		
τ_00__spatial lag_	0.05		
ICC	0.02		
Observations	69577		

^a^ Incidence rate ratios and intervals are back-transformed from the log scale.

^b^ Values in bold are statistically significant (*α*=0.05).

^c^ *Z*-value estimates for *post hoc* comparisons using Dunnett's test.

CI, confidence interval; ICC, intraclass correlation coefficient.

**FIG. 1.**
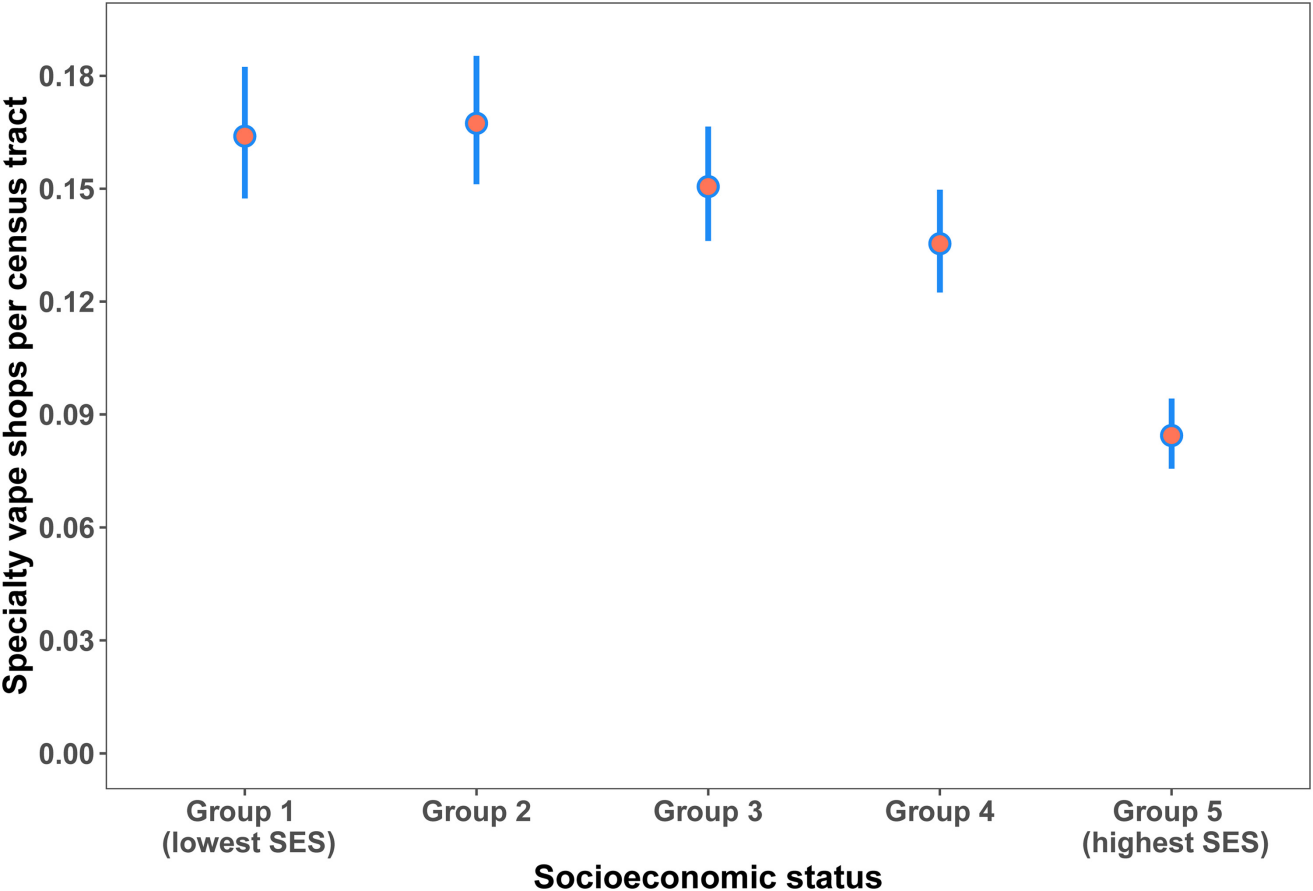
Specialty vape shop incidence, as a function of socioeconomic status, across the conterminous United States, 2018. The figure depicts model estimates (points) for socioeconomic quintiles and confidence limits (95% confidence intervals; lines) from a negative binomial generalized linear mixed-effects model. We back-transformed the values presented here from the original log link function-estimated model coefficients. *Post hoc* comparisons using Dunnett's test with socioeconomic status Group 5 (highest socioeconomic status) as the comparator group indicate statistically significant differences, and fewer vape shops, compared with all other groups (*α*=0.05).

As reported in Table 1, our results show a gradient in specialty vape shop incidence by SES. Multiple comparisons using Dunnett's test, with the highest SES (Group 5) and most privileged as the baseline comparator, indicated a significantly higher number of vape shops in all other SES quintile groups (Table 1 and Fig. 1). In particular, the two lowest SES quintiles (Groups 1 and 2) had nearly double the rate of specialty vape shop incidence compared with the highest SES quintile (Group 5) (Table 1 and Fig. 1).

**FIG. 2.**
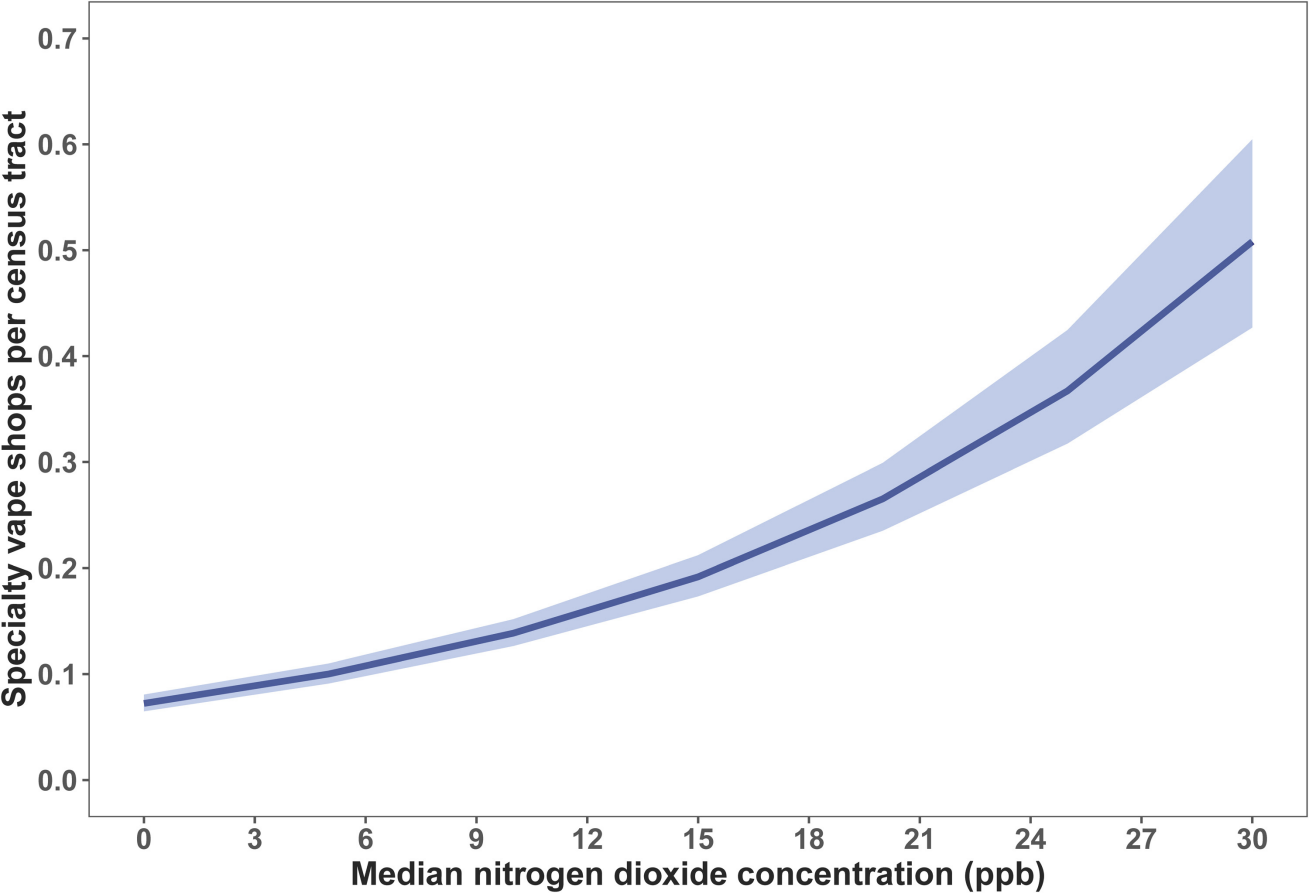
Relationship between specialty vape shop incidence and nitrogen dioxide concentration across the conterminous United States, 2018. Model estimates (line) and confidence limits (95% confidence interval; shaded area) from the negative binomial generalized linear mixed-effects model depict a significant positive association (*α*=0.05). We back-transformed the values presented here from the original log link function-estimated model coefficients.

Specialty vape shop counts were significantly positively associated with median NO_2_ concentration at the census tract level, adjusting for population differences. NO_2_ concentration increased by 7.00% in association with increasing vape shop incidence (Table 1 and Fig. 2).

## Discussion

The recent rapid increase in e-cigarette use prevalence among youth and young adults and the concomitant growth of the e-cigarette marketplace have raised public health concerns.^19–22,25^ To assess whether this marketplace may be contributing to the cumulative risks to which marginalized youth are exposed, we examined the association of specialty vape shop incidence with youth race/ethnicity, neighborhood SES, and air quality. Our results identify the disproportionate occurrence of specialty vape shops in low-SES and poor air quality neighborhoods. This is consistent with reports of disproportionate concentration of traditional tobacco retailers in low-SES communities.^3,5^

Our results did not identify disparities in vape shop incidence in relation to youth race/ethnicity at the census tract level. In this respect, the distribution of specialty vape shops differs from that of traditional tobacco retailers, which are disproportionately distributed in racial/ethnic minority neighborhoods.^5^

We believe our study is the first to characterize specialty vape shop association among youth with cumulative social stressors (SES) and environmental hazard attributes nationally. We identify disparities in vape shop incidence at the census tract level based on strict criteria for inclusion of vape shops, thereby controlling for confounding effects of tobacco retailers selling different products.^21^

This approach differs from previous national assessments that either include retailers that also sell other tobacco products^25^ or operate at the school district level.^21^ Employing a robust SES measure that indicates social advantages/disadvantages is a strength of our study. Previous national and regional assessments examining vape shop density association with neighborhood SES used one or few specific indicator variables (education, owner occupied housing, and income/poverty), with mixed results. For example, among national assessments, Dai et al.^25^ found no significant association of poverty and vape shops, while Venugopal et al.^21^ found a negative association.

Furthermore, due to our focus on cumulative risks experienced among youth, we examined race/ethnicity associations using census race/ethnicity data only for the population under the age of 18. At the census tract level, we found that the numbers of specialty vape shops decreased in relation to the proportion of youth represented by each of the race/ethnicity categories we examined. Particularly, we did not identify disparity in vape shop incidence in relation to youth race/ethnicity.

This result contradicts findings of previous national assessments that used race/ethnicity data for the overall U.S. population; these studies found positive associations between vape shop density and the proportions of racial/ethnic minority populations.^21,25^ The divergent results could be attributed to the higher racial/ethnic diversity among youth, with increased minority proportion, compared with the overall U.S. population.^34^

On the other hand, our findings are consistent with Dai et al.^25^ who reported a negative association of vape shop density with percentage of people under 18 years of age in urban areas.

Broadly, our results help contextualize socioenvironmental determinants that may lead to tobacco-related health disparities in relation to e-cigarette products and the retail marketplace. The disproportionate occurrence of both specialty vape shops and traditional tobacco product retail outlets in low-SES neighborhoods raises public health concerns. Furthermore, the inequitable distribution of specialty vape shops in low-SES and poor air quality neighborhoods raises EJ and health equity concerns.

Recent reports indicate that e-cigarette usage may be associated with higher likelihood of asthma and chronic obstructive pulmonary disease.^28–30^ The disproportionate distribution of specialty vape shops in low-SES neighborhoods may provide marginalized youth with greater access and exposure to e-cigarette products, marketing, and advertising, which may potentially result in greater use^17,19,20,25,26^ and disproportionate health impacts. In addition, chronic exposure to NO_2_ concentrations is associated with the development of asthma and increased susceptibility to respiratory infections, especially among youth.^37,38^

Particularly, lower-income populations in the United States are disproportionately exposed to outdoor, residential, average NO_2_ concentrations than higher-income populations.^9,10^ The increased incidence of vape shops in poor air quality neighborhoods, particularly in an urban context^21,25^ with increased traffic emissions,^37^ further points to potentially disproportionate impacts on disadvantaged populations due to cumulative social and environmental risks.

Our study results should be interpreted with several limitations in mind. We employed a cross-sectional design examining a highly dynamic retail sector using a dataset compiled before regulatory implementations, such as restrictions on certain flavored tobacco products, and the global COVID-19 pandemic. The race/ethnicity findings in our study are not reflective of multiracial youth who are not Hispanic or who belong to other racial/ethnic categories that we did not examine.

We excluded vape shops that sell other tobacco products along with e-cigarettes to reduce their potentially confounding influence on associations at the census tract level. These retailers are still sources of exposure to tobacco product marketing and sale, and our findings may not reflect their influence. In addition, 1.40% of the total specialty vape shops were not included in the census tract data for final analysis due to differences in SES composite index data availability.

We suspect this is a relatively minor factor that is unlikely to have affected results as the summary statistics of race/ethnicity and air quality data were similar between the reduced and the larger datasets. Finally, our study findings are limited in terms of drawing inferences about actual disparities in health outcomes; rather, our results are most useful for identifying socioenvironmental contexts and determinants that may potentially lead to tobacco-related health disparities.

Nevertheless, we provide an overall robust EJ assessment characterizing U.S. specialty vape shops as cumulative environmental health risk factors for disadvantaged populations.

Our results—the inequitable occurrence of vape shops in low-SES neighborhoods—provide context for understanding potential disproportionate impacts on disadvantaged communities, especially youth, which may result from the increased presence of e-cigarette retailers in their neighborhoods. This may include increased exposure to e-cigarette labeling and advertising and increased opportunities to purchase e-cigarettes.^17,19,20,26^

This finding may provide useful background information for the U.S. Food and Drug Administration's review of applications to market e-cigarette tobacco products (e.g., Pre-Market Tobacco Applications and Modified Risk Tobacco Product Applications), particularly in terms of impacts on marginalized populations.^49,50^ Our results may also inform postmarket reporting requirements related to advertising and promotion of e-cigarette tobacco products in retail settings, similar to those described in the market orders for General Snus smokeless tobacco products.^51^

As disadvantaged communities are disproportionately exposed to e-cigarette retailers, our results add to growing evidence on the e-cigarette retail environment as an appropriate locus for policies to improve equity.^19,20,26^ Retailer-focused strategies aimed at limiting youth exposure to e-cigarette labeling and advertising; preventing sales to minors; and limiting retailers in low-SES neighborhoods may reduce susceptibility and initiation and help prevent tobacco-related health disparities due to youth e-cigarette use.^4,17,26^

Our results may inform policy efforts, such as local ordinances, which limit tobacco retail licenses based on overall density of tobacco outlets to prevent disproportionate impacts on socially disadvantaged communities. Our results may also help target efforts to prevent youth initiation and curb youth vaping (e.g., health education curricula and public education campaigns) in socially disadvantaged neighborhoods.

Overall, our study provides a baseline characterization of how specialty vape shops contribute to the cumulative social and environmental hazards to which socioeconomically disadvantaged youth are exposed. Study findings can provide useful contextual information for regulators and may inform future research and local policy efforts aimed at reducing tobacco-related health disparities and promoting equity.

## Supplementary Material

Supplemental data

Supplemental data

Supplemental data

Supplemental data

## Data Availability

Data used for analyses and results are available in the Supplementary Data.

## Disclaimer

Publication of this article was supported by the U.S. Food and Drug Administration, Center for Tobacco Products. The findings and conclusions in this report are those of the authors and do not necessarily represent the official position of the Food and Drug Administration. This study and its findings are not associated with and have no bearing on the Food and Drug Administration Center for Tobacco Products' compliance inspection activities, including regulated establishment inspections.

No inspection data or inspection data sources from the Center for Tobacco Products were used to compile the specialty vape shop retailer establishment dataset used in this research. The mention of commercial products, their sources, or their use in connection with material reported herein is for reference only and not to be construed as either an actual or implied endorsement of such products by the Food and Drug Administration.

## Abbreviations Used

CI: confidence interval
EJ: environmental justice
GLMM: generalized linear mixed-effects model
NO_2_: nitrogen dioxide
SES: socioeconomic status
VIF: variance inflation factors
