# Supplementary material for: The Co-occurrence of Specialty Vape Shops, Social Disadvantage, and Poor Air Quality in the United States: An Assessment of Cumulative Risks to Youth
Source: Health Equity. 2022 Feb 25;6(1):132–41. doi: 10.1089/heq.2021.0151 (PMC8896168; doi:10.1089/heq.2021.0151)
Supplement: Supplemental data [file Supp_TableS1.docx]

**Table S1. Summary statistics of socioenvironmental attributes by number of specialty vape shops in census tracts**

|  | **Specialty vape shops per census tract** | | | | | |
| --- | --- | --- | --- | --- | --- | --- |
|  | **0 (N=63180)** | **1 (N=5566)** | **2 (N=720)** | **3 (N=93)** | **> 4 (N=18)** | **Total (N=69577)** |
| **Total population** |  |  |  |  |  |  |
| Mean | 4418.8 | 5172.5 | 5614.8 | 5657.1 | 5552.3 | 4493.4 |
| Range | 24 - 51536 | 182 - 70271 | 1088 - 25766 | 1294 - 15033 | 1352 - 20661 | 24.0 - 70271.0 |
| Quartile 1, Quartile 3 | 2933.0, 5491.0 | 3527.0, 6285.0 | 3715.2, 6657.2 | 4168.0, 6990.0 | 3293.8, 6081.8 | 2982.0, 5573.0 |
|  |  |  |  |  |  |  |
| **Children’s Race/Ethnicity** |  |  |  |  |  |  |
| *White alone, not Hispanic or Latino* |  |  |  |  |  |  |
| Mean | 514.5 | 610.6 | 666.2 | 613.1 | 493.3 | 523.9 |
| Range | 0 - 9635 | 0 - 11252 | 0 - 6396 | 16 - 2798 | 0 - 1349 | 0 - 11252 |
| Quartile 1, Quartile 3 | 164, 737 | 257, 825.8 | 295.8, 891.2 | 309, 776 | 145.2, 656.5 | 173, 746 |
|  |  |  |  |  |  |  |
| *Black or African American alone* |  |  |  |  |  |  |
| Mean | 144.9 | 143.2 | 161.2 | 185.7 | 77.9 | 145 |
| Range | 0 - 5360 | 0 - 4047 | 0 - 2367 | 0 - 1216 | 0 - 657 | 0 - 5360 |
| Quartile 1, Quartile 3 | 0, 164 | 0, 170.5 | 7, 202 | 4, 263 | 4.5, 91 | 0, 166 |
|  |  |  |  |  |  |  |
| *Hispanic or Latino* |  |  |  |  |  |  |
| Mean | 255.4 | 270.8 | 283.6 | 290.7 | 343.7 | 256.9 |
| Range | 0 - 11364 | 0 - 5745 | 0 - 3885 | 0 - 2168 | 0 - 2466 | 0 - 11364 |
| Quartile 1, Quartile 3 | 23, 292 | 40.5, 345 | 50, 356 | 57, 373 | 35.2, 414 | 24, 298.8 |
|  |  |  |  |  |  |  |
| *Asian alone* |  |  |  |  |  |  |
| Mean | 47.1 | 56.4 | 61 | 47.3 | 101.8 | 48 |
| Range | 0 - 3901 | 0 - 5369 | 0 - 1459 | 0 - 600 | 0 - 1318 | 0 - 5369 |
| Quartile 1, Quartile 3 | 0, 42 | 0, 57 | 0, 64 | 0, 43 | 2.2, 55.5 | 0, 44 |
|  |  |  |  |  |  |  |
| *American Indian / Alaska Native alone* |  |  |  |  |  |  |
| Mean | 9.8 | 8.7 | 11.4 | 12.7 | 49.9 | 9.8 |
| Range | 0 - 2484 | 0 - 890 | 0 - 1568 | 0 - 244 | 0 - 793 | 0 - 2484 |
| Quartile 1, Quartile 3 | 0, 0 | 0, 0 | 0, 0 | 0, 0 | 0, 0 | 0, 0 |
|  |  |  |  |  |  |  |
| **Socioeconomic status (SES)** |  |  |  |  |  |  |
| Group 1 (lowest SES) | 14859 (23.5%) | 1072 (19.3%) | 144 (20.0%) | 18 (19.4%) | 5 (27.8%) | 16098 (23.1%) |
| Group 2 | 12926 (20.5%) | 1250 (22.5%) | 159 (22.1%) | 28 (30.1%) | 5 (27.8%) | 14368 (20.7%) |
| Group 3 | 12354 (19.6%) | 1209 (21.7%) | 170 (23.6%) | 23 (24.7%) | 3 (16.7%) | 13759 (19.8%) |
| Group 4 | 11516 (18.2%) | 1206 (21.7%) | 149 (20.7%) | 15 (16.1%) | 5 (27.8%) | 12891 (18.5%) |
| Group 5 (highest SES) | 11525 (18.2%) | 829 (14.9%) | 98 (13.6%) | 9 (9.7%) | 0 (0.0%) | 12461 (17.9%) |
|  |  |  |  |  |  |  |
| **Median Nitrogen dioxide concentration (µg/m^3^)** |  |  |  |  |  |  |
| Mean | 8.2 | 8.7 | 8.8 | 8.7 | 9.5 | 8.3 |
| Range | 0.2 - 28.2 | 0.4 - 23.9 | 0.2 - 21.7 | 3.2 - 16.7 | 4.0 - 20.8 | 0.2 - 28.2 |
| Q1, Q3 | 5.0, 11.0 | 6.2, 10.8 | 6.6, 10.8 | 6.5, 10.9 | 6.1, 11.9 | 5.2, 11.0 |
|  |  |  |  |  |  |  |
