# Supplementary material for: The Co-occurrence of Specialty Vape Shops, Social Disadvantage, and Poor Air Quality in the United States: An Assessment of Cumulative Risks to Youth
Source: Health Equity. 2022 Feb 25;6(1):132–41. doi: 10.1089/heq.2021.0151 (PMC8896168; doi:10.1089/heq.2021.0151)
Supplement: Supplemental data [file Supp_TableS2.docx]

**Table S2. Candidate generalized linear mixed-effects models (GLMM) analyzing specialty vape shops as a function of children’s race/ethnicity and socioenvironmental factors.** All the candidate models contained log-link functions. We selected the best fit and parsimonious model (in bold) based on combination of Akaike Information Criteria (AIC) and Bayesian Information Criteria (BIC), with lower values indicating better fit.

| **Candidate models and response distributions** | **Degrees of freedom** | **AIC** | **BIC** |
| --- | --- | --- | --- |
| **Negative binomial GLMM** | **12** | **46786** | **46895** |
| Zero-inflated negative binomial GLMM | 13 | 46785 | 46904 |
| Zero-inflated Poisson GLMM | 13 | 46904 | 47014 |
| Poisson GLMM | 11 | 47405 | 47506 |
| Hurdle models via truncated Poisson | 13 | 48542 | 48652 |
