# Supplementary material for: The Co-occurrence of Specialty Vape Shops, Social Disadvantage, and Poor Air Quality in the United States: An Assessment of Cumulative Risks to Youth
Source: Health Equity. 2022 Feb 25;6(1):132–41. doi: 10.1089/heq.2021.0151 (PMC8896168; doi:10.1089/heq.2021.0151)
Supplement: Supplemental data [file Supp_TableS3.docx]

**Table S3. Results of multicollinearity test for the selected negative binomial generalized linear model analyzing specialty vape shops as a function of children’s race/ethnicity and socioenvironmental factors**

| **Variable** | **Variance Inflation Factor** | **Increased SE** | **Tolerance** |
| --- | --- | --- | --- |
| Race/Ethnicity of youth |  |  |  |
| *Black or African American alone* | 1.22 | 1.1 | 0.82 |
| *Asian alone* | 1.17 | 1.08 | 0.86 |
| *American Indian / Alaska Native alone* | 1.02 | 1.01 | 0.98 |
| *Hispanic or Latino* | 1.33 | 1.15 | 0.75 |
| Socioeconomic status | 1.31 | 1.15 | 0.76 |
| Nitrogen dioxide concentration | 1.37 | 1.17 | 0.73 |
